# Parental Values During Tracheostomy Decision-Making for Their Critically Ill Child: Interviews of Parents Who Just Made the Decision †

**DOI:** 10.3390/children12091115

**Published:** 2025-08-25

**Authors:** Haoyang Yan, Cynthia Arslanian-Engoren, Kenneth J. Pituch, Patricia J. Deldin, Sandra A. Graham-Bermann, Stephanie K. Kukora

**Affiliations:** 1 Department of Psychology, University of Michigan, Ann Arbor, MI 48109, USA; pjdeldin@umich.edu (P.J.D.); sandragb@umich.edu (S.A.G.-B.); 2 Department of Family and Community Medicine, Heersink School of Medicine, University of Alabama at Birmingham, Birmingham, AL 35233, USA; 3 Department of Health Behavior and Clinical Sciences, School of Nursing, University of Michigan, Ann Arbor, MI 48109, USA; cmae@umich.edu; 4 Department of Pediatrics, C.S. Mott Children’s Hospital, Michigan Medicine, Ann Arbor, MI 48109, USA; kpituch@med.umich.edu (K.J.P.); skkukora@cmh.edu (S.K.K.); 5 Department of Psychiatry, University of Michigan, Ann Arbor, MI 48109, USA; 6 Center for Bioethics and Social Sciences in Medicine, University of Michigan, Ann Arbor, MI 48109, USA

**Keywords:** shared decision-making, pediatric bioethics, home mechanical ventilation, chronic respiratory failure

## Abstract

**Background:** Pediatric tracheostomy decisions are challenging for clinicians and parents, especially when a child’s survival/neurodevelopmental outcome is uncertain. Better understanding of parents’ values over the decision period is crucial for clinical decision-making. **Objective:** To describe parents’ values during tracheostomy decision-making for their critically ill child and to identify opportunities to improve parent–clinician shared decision-making (SDM). **Methods:** We thematically analyzed 12 semi-structured interviews with parents who recently faced a tracheostomy decision for their critically ill child. Three study team members with qualitative expertise reviewed the transcripts, identifying key topics independently. A codebook was developed, and data were coded. Key research questions guided analysis, with findings iteratively reviewed by the study team. **Results:** We identified parents’ values at the three time points: when the decision was introduced, during their deliberations of it, and when the ultimate decision was made. Initially, parents resisted tracheostomy because it threatens normalcy. They valued proof of a need for tracheostomy and information with certainty. As certainty for tracheostomy increased over time, parents’ hope focused on reversibility of tracheostomy and improvement in normalcy compared to current status. They concurrently worried about practical issues such as emergencies, home care, and finances. Key considerations driving the final decision included best interest of the child, perceived benefits of tracheostomy compared to its downsides or other options, and potential for better quality of life and longer life. **Conclusions:** Parents’ dynamic values shifting with clinical uncertainty suggests opportunities to improve SDM by attending to parents’ individualized needs and managing expectations.

## 1. Introduction

Tracheostomy placement is increasingly performed on chronically critically ill children with diverse conditions [1]. Many tracheostomy decisions (especially those also considering mechanical ventilation) are preference-sensitive; both pursuing tracheostomy and not doing so may be reasonable options. Tracheostomy may be the only alternative to continued intubation, noninvasive breathing support, or palliative care, to facilitate long-term survival [2]. Because the diagnoses of underlying conditions and long-term prognosis are often uncertain [3], these tracheostomy decisions require reflection on the family’s values, goals, and preferences [4].

For these values-based decisions with uncertain outcomes, a shared decision-making (SDM) approach is recommended for clinicians and parents to discuss medical information and options in the best interest of the child and family [5]. However, clinicians and parents may hold different values and considerations [6,7] and clinicians may not be trained to properly communicate these decisions [8]. As a result, parents may experience decisional conflict and regret during and after decision-making [9,10]. Therefore, it is important to understand parents’ values (i.e., what they consider important and how much they find different outcomes acceptable), which can be lasting and fluid [11]. We have learned about parents’ values around tracheostomy decision-making in previous studies that examined parents’ perceptions of and experiences after tracheostomy placement [2,12,13,14,15,16] as well as parents’ and providers’ experiences with the tracheostomy decision process [14,17,18,19], including those focused on decision authority and communication challenges [20], the role of religion and spirituality [21], and caregivers’ need for decision support [22,23]. However, as these values were often recalled by parents, they may be biased by the time that had passed since the tracheostomy and their post-decision experiences, including their child’s ultimate outcome. How parents consider their values as they are facing these decisions and how this changes from the time the decision is introduced to the time the decision is made remains elusive. To address this knowledge gap, we explored parents’ values that inform SDM at distinct phases of pediatric tracheostomy decision-making.

## 2. Materials and Methods

A qualitative study was conducted using semi-structured in-depth interviews with parents of hospitalized children who were being considered for tracheostomy at a ~250 bed Midwestern quaternary children’s hospital in the United States between April 2018 and August 2019. This quaternary hospital provides the most advanced and specialized medical care to medically complex children, including intensive care in pediatric, neonatal, and cardiac units, as well as many disciplines of medical and surgical subspecialty care with pediatric surgery, otolaryngology, cardiac surgery, and neurosurgery, among others. The study was done at the University of Michigan and was approved by the medical school’s institutional review board. Our team consisted of researchers and clinicians with expertise in decision-making, clinical psychology, nursing, intensive/palliative care, and qualitative research.

### 2.1. Participants

A purposive sample of eligible study participants included parents (biological or legal guardian, ≥18 years old, English-speaking) who had recently faced a values-based tracheostomy decision (including the possible need for mechanical ventilation) for their critically ill child in the neonatal or pediatric intensive care units (ICUs) during the study data collection period. We excluded parents of children who needed tracheostomy for acute or temporary problems (i.e., anticipated future decannulation with typical neurodevelopment, causing care team to feel tracheostomy was in the patient’s clear best interest and ethically obligatory [24,25]), or for whom tracheostomy was not in the clear best interest of the patient (i.e., tracheostomy would not achieve care goals such as prolonging survival and would be considered potentially non-beneficial [26] by the care team).

To recruit study participants, we contacted the ICU medical teams on a regular basis to ask whether there were critically ill children for whom a tracheostomy decision had been discussed with parents and a decision had just been made. A unit-based research coordinator approached the parents and asked for their consent to be contacted by the study team. Potential participants were told that the study team sought to better understand the decision processes of parents of critically ill children considering a tracheostomy placement. Once parents agreed, the study team contacted them to set up a time for a one-hour in-depth interview. All parents approached agreed to be contacted by the study team. A total of 17 parents were interviewed. We aimed to contact parents within a week of their tracheostomy decision. All interviews occurred within 3 months of the tracheostomy procedure (for those who chose tracheostomy) or a recent discussion (for those who were not in favor of tracheostomy); 14 of the 17 interviews were conducted in three weeks or less.

### 2.2. Data Collection

Clinical psychologists and research staff trained in qualitative interview methods conducted the semi-structured interviews. Interviews (about 30–60 min) were conducted in-person (*n* = 12, in the hospital) or via phone (*n* = 5) based on mutual availability of parents and interviewers (one clinical psychologist and one research staff). At the beginning of each interview, written informed consent was obtained for participation, audio recording and for obtaining information from the child’s medical record (demographics and diagnosis). Participants were asked to provide self-reported demographics and received an honorarium (USD 50 gift card) for their participation. Interviewers used an interview guide (Appendix A) developed in iterative team meetings by our research team with decision science, qualitative methods, and clinical expertise based on the Cardinal Issue Perspective (CIP) of decision-making [27]. Built upon research from diverse fields (e.g., psychology, health care, management), the CIP proposed 10 Cardinal Issues every decision maker must consciously or unconsciously address to arrive at a decision. This comprehensive conceptual framework examines and explains successes and failures in decisions [27]. It has been used to examine other high-stakes decisions such as elder persons’ participation in health care decisions [28], and it may also apply to SDM in end-of-life situations [29].

The semi-structured interview guide was iteratively adjusted and revised after the first five interviews based on input from parents to better capture parents’ perceptions of values and the decision-making process. In the initial interviews, some language used in the questions was confusing to parents, and some aspects were not pertinent to their decisions. For example, questions were added to elicit parents’ emotions when making the decision, perceptions of most valued treatments’ upsides and downsides (i.e., benefits and risks), tradeoffs, interpretations of quality of life and child’s best interests, and regret. Starting with the sixth participant, a verbal summary of responses was provided at the conclusion of the interview along with the opportunity to correct and refine the summary to accurately reflect their experience. Data collection using the revised interview guide ended when data saturation was reached (i.e., redundancy and no additional information emerged during data collection).

### 2.3. Data Analysis

Due to the addition of interview questions, the first five interviews were considered to be pilot interviews. The final analysis included twelve interviews. Each audio-recorded interview was transcribed verbatim and checked by three undergraduate research assistants and the first author (H.Y.) to ensure accuracy and completeness.

We summarized demographic and medical information using descriptive statistics and analyzed qualitative data using thematic analysis [30]. Two team members with expertise in qualitative analysis (H.Y. and C.A.-E.) independently read all the transcripts multiple times to familiarize themselves with the data and denoted the transcripts with important ideas and possible codes. A third experienced qualitative researcher, a practicing neonatologist (S.K.), read three transcripts and identified important ideas, which agreed with those generated by the two primary coders. After the development of a codebook, one researcher (H.Y.) sorted the data into each code category. Two team members (C.A.-E. and H.Y.) reviewed and refined the coding (e.g., combine some categories) to prepare for analysis. What parents felt important at three stages was iteratively analyzed for clinical implications based on input from S.K and the team reached consensus. Quotes were reviewed for accuracy and completeness.

## 3. Results

Parents ranged in age from 19 to 63 years (median 31.5 years, all self-identified as female and three self-identified as non-White). Most were unmarried and reported being Christian/Catholic/Baptist, with diverse ranges of education and income. (See Table 1 for parent and child characteristics.) Because we recruited from the ICUs, these pediatric patients had prolonged hospital stays prior to tracheostomy decision-making faced by their parents. The majority of them were intubated at the time of decision-making, while a few were on respiratory support higher than what could be safely achieved for home-going without tracheostomy/mechanical ventilation. They had failed attempts to wean support previously. For one patient, final decision-making took place while the patient was in the operating room due to anticipated long-term respiratory support needs.

**Figure 1:**
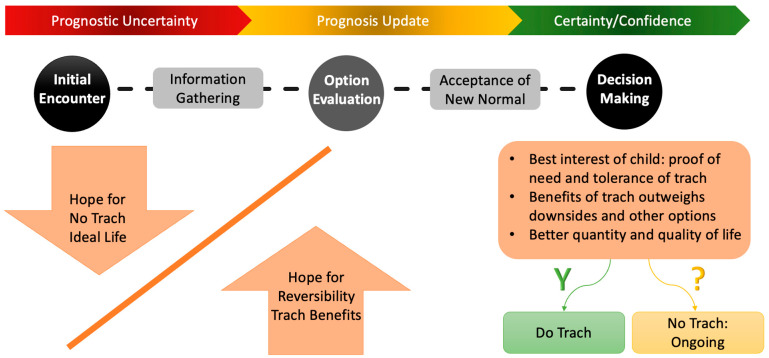
Dynamic values in decision-making.

Three themes with subthemes describing parents’ values throughout the decision-making process were identified (see Figure 1).

**Theme 1: Values in initial encounters:** against the invasive nature of tracheostomy, hope for no tracheostomy, proof of child’s need for tracheostomy, need for reducing uncertainty, sufficient time to consider (for quotes, see Table S1).

When parents initially learned about the tracheostomy option, most immediately resisted it for several reasons. First, tracheostomy was a new concept that they had “never heard of” and found “scary”. Parents perceived it as invasive, therefore instantly not wanting to think about it. Parents kept asking for other options with the hope that they could “avoid” it (e.g., if the child could wean off or grow stronger to breathe on their own). The nature of tracheostomy and complex situations with reactive emotions (“head was not there”, “sad”, “devastated”, “hopeful”, “fear”) made it difficult for parents to engage in the conversation about this option with doctors.

Second, parents did not always accept the clinical indications for tracheostomy their medical team presented when uncertainty remained. Parents wanted “to be shown” evidence that their child “really needed” tracheostomy. Parents reported dismissing the idea of tracheostomy before the child’s intubation or when they felt there was insufficient “proof”. Only one parent described having “no hesitation” to tracheostomy because they felt their child could tolerate it. Parents reported that, initially, their clinicians’ communication was ambiguous. Their doctors did not say that tracheostomy was needed or include multidisciplinary teams. Parents saw this ambiguity and uncertainty as an opportunity to be hopeful or avoid earnest consideration. While this hope was important, parents reflected that having more definitive advice and information from the medical team would have helped them more seriously consider tracheostomy “in the beginning” and feel more prepared when the time for decision approached to make the “right” decision, e.g., after unsuccessful weaning from respiratory support.

Following parents’ initial denial, they focused on questions and worries: How will my child respond to tracheostomy? How will life change? Parents began seeking additional information at this point. It took parents a few discussions to accept the idea of tracheostomy, and even then, they did not want to make a rushed decision. Parents who did not get the chance to have multiple conversations and sufficient time to consider their decision reported “want[ing] some more time”. One parent faced with an immediate decision when their child was in the “operating room” described the importance of trusting the doctor, because they “didn’t have that luxury” to do research and think more deeply, although “tracheostomy has always been hanging out there”.

**Theme 2: Values when weighing options:** survival, normalcy, development, better quality of life, reversibility, safety, home care, family, and social environment (for quotes, see Table S2).

When evaluating the tracheostomy option, parents described important benefits of tracheostomy. Parents valued their child’s “normalcy” and having a “better quality of life”, which were idiosyncratic. For example, parents described “a secured/stable airway” and more “comfort” in breathing posed an improvement over the constant uncertainty that a possible life-threatening respiratory event could occur. Breathing more easily would allow the child to reserve energy for self-development, such as to “eat”, “drink”, and “sit up”. It would also allow the child to be active and interactive, instead of being intubated. Parents highlighted the value of their child being able to receive more treatments. Being able to “come home” and “get out of the hospital” was another important indicator of normalcy for the family and for the child. This also posed a long-term possibility for the child to have “normal life” experiences like other children, such as to “make friends and go to school”.

On the other hand, parents expressed several concerns about tracheostomy that weighed heavily in their decision-making. First, whether their child’s tracheostomy would be permanent was among the biggest concerns. Parents universally hoped that the “tracheostomy would be temporary” and that their child could be decannulated. They expressed that though it created some normalcy compared to their current condition, tracheostomy itself and life-long ventilatory support was “not normal” or desired in the long term. Second, the risks of airway emergency and infection created fear in parents. Related to their worries about their child being harmed by a serious event, they also worried about their own roles and responsibilities in preventing harm from befalling their child. Third, parents described concern about tracheostomy prohibiting specific activities they considered important. Their child may not “be able to talk”, to socialize, to participate in activities such as “swimming”. Fourth, parents described concerns about how tracheostomy management would impact life. Caring for the child requires in-home nursing care, which caused “fear” and worries about bearing “financial strain”. Parents had concerns that the family’s life would be impacted as well, such as not being able to take vacations, or the care needed by their one child deterring attention from their other children. Parents also described dreading unwanted sympathy and rude comments from strangers.

**Theme 3: Values driving the decision:** best interest of child including proof of need and tolerance of tracheostomy, benefits of tracheostomy outweigh its downsides or other options, better quantity and quality of life (for quotes, see Table S3).

In our sample, 11 of 12 parents came to accept and decide to pursue tracheostomy, though they still hoped that tracheostomy would not be needed right up until it was placed. They reported that doctors’ reassurance that “it can be reversed” was a key factor in their acceptance.

Parents reported that concluding that their child really needed tracheostomy to get better gave them clarity in the decision-making process. Parents described having to stifle their emotions (e.g., “sadness”, “grief”) and deprioritizing their own feelings and wishes to consider their child’s needs more objectively. Parents acknowledged that tracheostomy would bring more responsibilities, and their “convenience” would be sacrificed, so they had to convince themselves of this reality. Ultimately, the final decision was based on “best interest” and “love” for their child. The parent whose child did not undergo tracheostomy also suggested had her child showed proof that it was necessary, she would do it.

Although parents had to give up their ideal expectation for their child’s life, they perceived that the benefits of tracheostomy outweighed its downsides. Parents weighed short-term risks and challenges against the possibility of best long-term benefits. They perceived tracheostomy giving their child “a chance at life” to “thrive”, while expressing that they were willing to take the risks only if they felt confident their child could tolerate and survive the tracheostomy. Parents indicated that they would not put their child through tracheostomy if it would result in “pain”, “suffering”, or their child would not be able to “experience” the world.

The benefits of tracheostomy also outweighed other options (intubation, jaw distraction, extubation, comfort care). For example, when their child was intubated or would be intubated if jaw distraction were performed, parents weighed the risks (e.g., damaging vocal cords with intubation) against benefits of tracheostomy, in particular “going home” and leading a “normal” life. Parents were reluctant to have their child undergo additional procedures and expressed that they did not want the child to experience further suffering if another extubation attempt or surgery failed. Compared to comfort care, one family felt their child’s life ending was not acceptable, so they chose tracheostomy. One parent indicated that the decision depended on which option would bring “comfort and peace” to their child. Although some parents reported valuing survival more than quality of life or vice versa, many parents had difficulty “separate[ting] these two”. Tracheostomy was chosen because these parents felt it could extend life and improve their child’s quality of life (at least “be more comfortable”).

## 4. Discussion

For tracheostomy decisions, like most complex medical decisions in pediatric patients, the patients themselves do not have decisional autonomy, and surrogates must make these decisions based on the medical situation and considerations of the patients’ best interest and their presumed values. For decisions where there is not a clear “right” answer, parents are generally the legal and ethical surrogate decision makers, and decisions should be based on their values. Disagreements and conflicts between parents and the medical team may arise in high-stakes decisions, sometimes based on values differences around the acceptability of the possible outcomes [31,32]. A growing body of literature has focused on understanding parents’ values in complex medical decisions for their children [32,33,34,35,36,37,38,39]. These have noted parents’ decisions are influenced by changes in their child’s condition, input from extended family and community, their prior knowledge and experience, and personal factors, such as emotions and faith/spirituality.

Our study explored parents’ experiences with SDM around tracheostomy immediately following the decision, but before their perceptions could be influenced by the passage of time and their child’s outcome. It offers insights into parents’ dynamic expression of values (throughout tracheostomy decision-making). We noted several findings with important implications for clinical communication.

Initially, parents were resistant to the option of a tracheostomy. However, for some this changed over time, as additional prognostic information from the medical team led to acceptance. Parents preferred not to be rushed to make the decision, a finding consistent with previous studies in other patient populations that noted insufficient time led to worse decision-making experiences [40]. Thus, the downsides of additional uncertainty and consequent parent resistance with early introduction of the possibility of tracheostomy must be weighed against the benefits of allowing parents more time to consider the decision and possible options. Previous tracheostomy SDM work reported the complexity of discussing uncertainty in this decision [18], but did not specifically describe how clinicians’ communication around uncertainty as its changes over time can influence parents’ acceptance of the decision. Our findings highlight the need for early, repeated, and clear communication of the potential indications and expected outcomes with tracheostomy, along with a timeline for additional evaluation and eventual decision-making. Managing expectations and delivering a consistent message is an important premise of SDM [41].

We also found that parents’ changing perceptions of uncertainty were closely intertwined with hope. When parents’ hopes that their child could avoid tracheostomy diminished, their hopes shifted to their child being eventually decannulated as prognostic certainty increased over time. For parents of children with complex illness, hope is not only important for coping and resilience, but plays an active role in how parents consider decisions and interact with clinicians [21,32,42,43]. This suggests that SDM between clinicians and parents should support parents’ hopes and, when specific hopes become unrealistic with additional time and information, assist parents in re-framing their hopes to align with achievable goals. Parents’ hopes and values often centered around “normalcy”, though they endorsed differing beliefs around what this meant for their child and family. Achievement of developmental milestones and being able to go home—hopes that parents expressed once they had accepted tracheostomy—align with previous studies that described parent-reported child-centered outcomes after a tracheostomy placement [2] and that parents value opportunities to improve their children’s capabilities [44,45]. Our findings further support the importance of clinicians understanding and respecting that families have different values and perspectives in decision-making [46,47], focusing on transparent communication around risks and benefits of tracheostomy [48] and engaging in realistic discussions of long-term implications for child and family [16,37,49,50].

Parents mentioned considering the practical and social impact of tracheostomy when weighing options. This is important information to have during decision-making [14], which may help address parents’ worries and prepare them for future challenges, though it does not appear to be the determining factor in our study sample. For instance, many of the parents interviewed in our study identified as unmarried, though several indicated that there was a partner/co-parent involved in the care of the child and in the decision-making process. The complex post-discharge care needed for patients with tracheostomy necessitates at least two trained caregivers who may respond in emergencies, and that the child remains in the care of these caregivers or skilled home nursing clinicians at all times, resulting in caregiver burden and challenges [12,51]. For this reason, decisions about tracheostomy for these families were likely more nuanced due to social circumstances. Despite this, the majority moved forward with tracheostomy, and this consideration was slightly discussed in the interviews of these parents, suggesting that while family and social support likely weighs in on these decisions, this is not the factor most strongly prioritized by parents.

Parents most deeply considered the best interest of their child and family when making the ultimate decision. This is consistent with previous findings of parental decision-making for tracheostomy [20] and aligns with the literature on “good parent” beliefs [34,37,52]. All parents in our study felt that quality of life was paramount, though they stressed extending life on different levels. We found that parents did not see quantity and quality of life as mutually exclusive. Parents emphasized that they would not pursue tracheostomy just to prolong life if their child’s quality of life were what they considered unacceptable. While some studies on goals of care decisions have noted that some parents may value survival at all cost [6], others have identified that quality of life is always considered by parents, who find a wide range of possible outcomes acceptable [31].

Interestingly, for parents who chose tracheostomy, it was a final and definitive decision, while for parents who declined tracheostomy, it remained an ongoing decision. This raises several interesting questions whether, and on what timeline, a decision to decline tracheostomy should be readdressed. Parents may need to reconsider their values as the child’s current medical condition changes. Concurrently, clinicians should be cautious not to pressure families to choose tracheostomy against their wishes due to misperceptions that the team’s persistence signifies it is the “right” or “recommended” thing to do. Further study is warranted to understand parents’ preferences for continued discussion about tracheostomy for their child when they have initially chosen not to pursue this intervention.

Our study has both limitations and strengths. First, while there were only twelve interviews analyzed, we achieved thematic saturation consistent with recommendations for qualitative research [53,54]. Overall, the number of parents who make preference-sensitive pediatric tracheostomy decisions is low, even in large quaternary referral centers. Facing these decisions while also parenting a critically ill child is emotionally stressful, and approaching them in a sensitive and timely way to discuss their experiences can be challenging. We strove to interview parents as soon as they felt comfortable sharing their tracheostomy decision-making, thereby reducing recall and outcome bias for capturing important perspectives necessary for clinical practice. Additionally, there were few parents who had chosen against tracheostomy. This may be because there are many decision-points for goals of care for critically ill children, and many parents who would have found life-sustaining treatments unacceptable opted for comfort care before a thorough evaluation for tracheostomy. Future studies of parents facing other goals-of-care decisions during their child’s acute illness in the ICU may help shed light on the perspectives of these parents. We did not capture parents of patients who were not on respiratory support at the time of decision-making and were considering tracheostomy as an elective or prophylactic intervention, choosing instead to focus on critically ill patients in ICU facing these decisions. Additional studies of parental decision-making under these different circumstances are warranted in the future. Lastly, all of our participants were female, English-speaking, and mostly religious (Christian/Baptist/Catholic) and the study was conducted at a single center. While this reflects the demographics of our center and scope of the study, a more diverse sample is needed to understand more nuanced and different perspectives. Nonetheless, our participants provided rich narrative data that described a variety of experiences and saturation was reached. Data were validated by the opportunity for parents to add to, confirm, and correct interviewers’ verbal summary of their responses at the end of the interview. Analysis was conducted by a research team with diverse backgrounds and experiences, heightening reflexivity of the analysis, interpretation, and findings.

## 5. Implications and Conclusions

The study has implications for patient-centered practice and future research. Our findings demonstrate the dynamic and evolving process of tracheostomy decision-making. Increasing understanding of parents’ experiences with distinct stages in the SDM process can assist clinicians in engaging parents and eliciting their values and preferences. Our research further supports opportunities to improve SDM by exploring the plurality of values parents hold when considering their child’s future with or without a tracheostomy. Clinicians should be attentive to how some of parents’ values adapt over time while some remain fundamental. It is also important for clinicians to approach this process with patience and prepare both oneself and parents for the next stage of decision-making. The goal is to facilitate SDM on a timeline that encourages parents to be adequately informed and engaged in decision-making but does not cause harm to the patients. Further research is warranted to examine how approaches to SDM for pediatric tracheostomy in practice can lead to increased parental satisfaction regarding the decision process and its outcomes.

## Figures and Tables

**Table 1:** Parent and child characteristics.

Characteristics	Value *
**Children whose parents participated in interviews (*n* = 12)**	
Age, median (range)	6 months (2 months–14.6 years)
Sex, *n* (%)	
Male	8 (67)
Female	4 (33)
Primary diagnostic categories, *n* (%)	
Genetic conditions	5 (42)
Prematurity/Bronchopulmonary dysplasia	3 (25)
Brain injury/malformation	3 (25)
Heart conditions	1 (8)
Decision at the time of the interview, *n* (%)	
Tracheostomy	10 (83)
1. Tracheostomy already placed	9 (90)
Time between tracheostomy placement and interview (range)	1 day–approximately 3 months
2. Before tracheostomy placement	1 (10)
Time between interview and tracheostomy placement	6 days
Pending/No tracheostomy	2 (17)
Time between discussion and interview (range)	Ongoing/2–3 months
**Parents who participated in interviews (*n* = 12)**	
Age, median (range)	31.5 yrs (19–63 yrs)
Relationship to the child, *n* (%)	
Biological mother	11 (92)
Legal guardian (female)	1 (8)
Race, *n* (%)	
White	9 (75)
Black or African American	3 (25)
Religion, *n* (%)	
Christian/Catholic/Baptist	9 (75)
None indicated	1 (8)
Rather not say	1 (8)
Missing	1 (8)
Marital status, *n* (%)	
Married	4 (33)
Unmarried (including other: 2 noted single, 1 noted engaged)	8 (67)
Education, *n* (%)	
Some or no high school	3 (25)
High school	1 (8)
Some college	3 (25)
College graduate	3 (25)
Master’s degree or higher	2 (17)
Household income, *n* (%)	
<USD 30,000	2 (17)
USD 30,000–USD 50,000	1 (8)
USD 50,000–USD 90,000	4 (33)
>USD 90,000	1 (8)
Rather not say	4 (33)

* To date of the interview.

## Data Availability

The original contributions presented in this study are included in the article/Supplementary Material. Further inquiries can be directed to the corresponding author.

## Supplementary Materials

The following supporting information can be downloaded at: https://www.mdpi.com/article/10.3390/children12091115/s1, Table S1. Illustrative Quotes of Parents’ Values in Initial Encounters (Theme 1). Table S2. Illustrative Quotes of Parents’ Values when Weighing Options (Theme 2). Table S3. Illustrative Quotes of Parents’ Values Driving the Decision (Theme 3).

## Appendix A

**Interview Questions**

1. **[Need]** How did this decision of whether to have your child undergo a tracheotomy first come up?

Probe: Was it changes in the child’s condition? If so, what were those changes?

2a. [Mode—Who]

2a (1)a. Who participated in discussions, in the hospital setting, about whether your child would undergo a trach? For instance, a family member, a health care professional, a religious leader, a friend, and/or other parents?

2a (1)b. Why were they included in the discussions about whether your child would undergo a trach?

2a (1)c. Were there any discussions about this decision OUTSIDE the hospital setting? If so, who participated in discussions about making the decision? For example, a family member, religious leader, a friend, and/or other parents?

2a (1)d. Why were they included in the discussions about whether your child would undergo a trach?

2a (2)a. We would like to know where on the physician-directed decision/shared decision/parent-family decision spectrum the decision was made? Imagine a line from 0 to 100, with physician-directed decision on the left (0), parent-family decision on the right (100), and shared decision in the middle (50), what number from 0 to 100 do you think best represents how this decision was made?

2a (2)b. Why did you choose that number?

2b. [Mode—What/How]

2b (1)a. During the time the team and family were contemplating the trach, did you consult with other people as to whether to do the trach? For example, other providers, other families.

2b (1)b. Whom in particular did you seek?

2b (1)c. What in particular did you find?

2b (2)a. Did you consult any informational resources as to whether to do the trach? For example, decision aids, web sites or journals, materials?

2b (2)b. What in particular did you seek?

2b (2)c. What in particular did you find?

3. **[Investment]** Next, let’s turn to the efforts you took to make the trach decision. The efforts could include time and other costs.

3a. [Investment—Time]

3a (1). How many discussions regarding the trach decision did you have with health care professionals (including doctors, nurses, clinical social workers)?

3a (2). How much time in total do you think was spent on the discussions with the health care professionals?

3b. [Investment—Emotions/Stress]

3b (1). What emotions or feelings did you experience during decision making?

3b (2). How difficult was it for you to manage any stress and other unpleasant emotions that might have arisen when you were thinking through the trach decision for your child?

Not Difficult at All/Somewhat Difficult/Highly Difficult/Extremely Difficult

3b (3). What actions did you take to deal with the stress and other unpleasant emotions that arose when you were thinking through the trach decision?

4. **[Options]** Next, let’s turn to possible options for your child’s condition.

4a. [Options—Awareness]

4a (1)a. First, did you feel that there was actually a choice? (Why did you feel that way?)

4a (1)b. Were there any other non-trach option discussed?

4a (2). Sometimes medical professionals use the term “comfort care” to mean medical care that focuses on relieving symptoms, optimizing comfort, and improving quality of life. Was comfort care ever discussed?

4b. [Options—Discovery/Generation] As best you can remember, how did this option come up?

U5. **[Possibilities]** Now let’s consider possible outcomes of pursuing this option, both the positive (“upsides”) and negative (“downsides”). Let’s talk about upsides first.

U5a. [Possibilities—Specific]

U5a (1). What did you think were the most significant potential “upsides” for this option and why? Upsides would include benefits and hopes.

U5a (2). Which of these upsides was particularly important to you? Why? **[Value]**

U6. **[Chances]** Please tell us what you saw as the chances that each of those upsides for this option would actually happen—either None at All, Very Low, Somewhat Low, Somewhat High, Very High, or Guaranteed.

U6 (1). What did you see as the chance that ______ would actually happen?

U6 (2). When you said _____ (None at All, Very Low, Somewhat Low, Somewhat High, Very High, or Guaranteed), what probability number between 0% and 100% do you think best describes what you meant?

D5. **[Possibilities]** Now let’s talk about downsides of this option.

D5a. [Possibilities—Specific]

D5a (1). What did you think were the most significant potential “downsides” for this option and why? Downsides would include risks, fears, and worries.

D5a (2). Which of these downsides was particularly important to you? Why? **[Value]**

D6. **[Chances]** Please tell us what you saw as the chances that each of those downsides for this option would actually happen—either None at All, Very Low, Somewhat Low, Somewhat High, Very High, or Guaranteed.

D6 (1). What did you see as the chance that _____ would actually happen?

D6 (2). When you said _____ (None at All, Very Low, Somewhat Low, Somewhat High, Very High, or Guaranteed), what probability number between 0% and 100% do you think best describes what you meant?

7. **[Value]**

7a (1). What do you think your child’s best interest (or goals of care) is in the context of this decision?

Probe: (if the parent hints something different for the child and the family) What do you think your or your family’s best interest is in the context of this decision?

7a (2). What does best quality of life for your child mean to you in the context of this decision?

7b (1). Are there any principles that guided you in the making of this decision? If so, what is it?

8. **[Tradeoffs]** One of the reasons that trach decisions are difficult is that there may not exist a clear and certain “best” option. Often, none of the options is able to satisfy all needs and free from unwanted outcomes. As a result, parents often have to consider tradeoffs between options. What I mean by tradeoffs is that all options have both pros and cons, and one has to give up something in order to get something else.

8a. From your perspective, what are the core tradeoffs of the trach decision?

8b. In the end, how was the final decision made?

9. **[Acceptability]** Who were the people—for instance, family members, friends, community members, or health care professionals (colleagues, nurses, social workers)—whose happiness or unhappiness might be influenced by the trach decision for your child? For instance, some relevant people might agree with the decision while some might disagree.

9a. [Acceptability—How]:

9a (1)a. In the end, did you feel that there was agreement among everyone involved in the decision? If so, how did that happen?

9a (1)b. If not, how did you deal with the feelings and potential actions of those individuals?

10. **[Implementation]** Once the decision was made about whether your child would have a trach, what happened next?

11. **[QOL]** People have different values and different ways of being a good parent when making these kinds of decision. Some parents want the longest life possible for their child. Some worry more about the quality of their life more than the quantity of their life.

How did your feelings about valuing quality vs. quantity of life play a role in the decision?

12. **[Regret]**

12a. Thinking back, what information did you wish you had gotten or what did you wish you had done for making this decision?

12b. Do you have any regret regarding the final decision? Why?

**[Confirmability]** The interviewer summarizes the participant’s responses to all questions above and asks, “Did I get your story and opinions right? Is there anything that I missed?”

13. Are there other things that informed or influenced this decision that we have not asked about?
