# Supplementary material for: Parental Values During Tracheostomy Decision-Making for Their Critically Ill Child: Interviews of Parents Who Just Made the Decision
Source: Children (Basel). 2025 Aug 25;12(9):1115. doi: 10.3390/children12091115 (PMC12468954; doi:10.3390/children12091115)
Supplement: Supplementary file 1 [file children-12-01115-s001.zip › children-3722648-supplementary.pdf]

Supplementary Materials

Table S1. Illustrative Quotes of Parents' Values in Initial Encounters (Theme 1)	
Against the invasive nature of tracheostomy	"Of the whole decision of the trach, I'm like oh my god you have to cut my baby's throat." (C10, trach, < 1 week after placement)<br><br>"It was a very hard decision for me. Cause I never heard of it and I heard that it's painful." (C14, pending/no trach yet, ongoing discussion)
Hope for no tracheostomy	"My head was not there. Cause really my prayers were I was against the trach." (C6, trach, 2-3 weeks after placement)<br><br>"At first it was kind of almost like a denial like they'll find something else, cause this has been on a conversation for like a week like they'll, and then they keep bringing up other options so it's like there's no way she'll get a trach, they'll find another way to do this." (C9, trach, < 1 week before placement)<br><br>"Trach was always a thought that was in there, it was a really—at that point, it was a really scary thought, like, oh, I really hope we can avoid a trach that was always my, oh whatever we can do, I just hope we can avoid a trach." (C11, trach, < 1 week after placement)
Proof of child's need for tracheostomy	"The first time was before he got intubated and I didn't want it of course. The second time we were talking about his care plan and I DID NOT want it again…The third time he was already intubated and it was either stay intubated and risk losing his vocal cords or go ahead and do the trach…" (C7, trach, 1-2 weeks after placement)<br><br>"Just wanting proof, just wanting to be shown something that says that he's—he can't do it. The trach is what's best. I wanted something to show me that the trach is what was best. So, I kept waiting for him to have a hard time with a wean. Or, he stopped gaining weight because he's spending too much calories trying to breathe. Or his labs were getting funky because he was unable to whatever, something. I needed some kind of something that told me that he needed a trach, not just because he passes certain spot where they say, ok, well, he's gonna need a trach." (C15, definitive no trach, 2-3 months since discussion)<br><br>"She was showing that she was a fighter. So, we knew this was the best decision for her." (C8, trach, 1-2 weeks after placement)
Need for reducing uncertainty	"In the beginning... there could have been more communication about if they [doctors] really thought that she needed that. He [doctor] didn't say this has to be done. Maybe it would have helped if he did say that…just so my head could be there." (C6, trach, 2-3 weeks after placement)<br><br>"I wish they [pulmonologists] would have been invited into the party sooner in helping us with that information. I don't know if we would have changed, I don't know if we would have hung out for 5 months but maybe we would've still held on a little longer and came up with an estimate of the likelihood, how many other kids have done this. Like how can we best make this decision if we understood his severity." (C13, trach, < 1 week after placement)

Supplementary Materials

	"Probably the unknown…I really didn't know how it worked. It just seemed very intimidating at that point…It was the feeling of fear of making the wrong decision. Overwhelmed, because both options really weren't great options, and we didn't really want to make it either one. We just wanted it to fix itself." (C11, trach, < 1 week after placement)<br><br>"I will try not to cry…I was devastated…finding out that he wasn't just, a preemie baby that he was supposed to…he was going to be a trach baby was even harder. It was very scary…and especially since there's really no right or wrong…like what to do…" (C15, definitive no trach, 2-3 months since discussion)
Sufficient time to consider	"I'm afraid of how it would work and how it would move. It was just a lot of questions." (C14, pending/no trach yet, ongoing discussion)<br><br>"We didn't wanna make a decision so fast when we wasn't sure about it." (C16, trach, 3 months after placement)<br><br>"I was sad at first, I never had to go through this. I sat and thought about it for a couple days…I did want some more time but then I had to go back at the work too at the same time. I work Monday through Friday sometimes Saturday, so I got to go with the trach, cause he'll come home soon." (C17, trach, 2-3 weeks after placement)<br><br>"Probably about 20 minutes, because he was in the OR and we didn't have that luxury." (C12, trach in OR, 1-2 weeks after placement)

Supplementary Materials

Table S2. Illustrative Quotes of Parents' Values when Weighing Options (Theme 2)	
Important benefits: survival, normalcy, development, better quality of life	"He would be breathing. We don't have to worry about that. That's ultimately so important." (C12, trach in OR, 1-2 weeks after placement)<br><br>"I'd have to say the most important one would be a guaranteed airway, but I think the most important one to me and my emotional state specifically is what I want for [patient] is to grow as a healthy normal child and I think the playing and the growing is personally my more important thing." (C9, trach, < 1 week before placement)<br><br>"So [doctors] did make us aware that she would be more, that she would move forward. That she would have a life like other kids and be able to do things instead of just laying there with a tube down her throat. So that was positive to hear. That she could be carried and loved." (C6, trach, 2-3 weeks after placement)<br><br>"Being able to crawl and sit up and you can do that better at home and be part of our family like right now it's like my life here and my life there and they come and visit sometimes, but it's still mom's life in the hospital with [patient] so having the trach gets us all into a family home." (C13, trach, < 1 week after placement)<br><br>"That's the big thing right there, really getting him home." (C17, trach, 2-3 weeks after placement)<br><br>"Having as normal of a life as possible for him. He's not even five. I want him to be able to go to school, I want him to have friends, I want him to be able to run and talk and play. Of course I don't want him to go through all of the stuff he's going through. But it's something that will help him have a normal life that will help him make friends and go to school." (C7, trach, 1-2 weeks after placement)
Important downsides: reversibility, safety, home care, family, and social environment	"The only downside I had with making the trach decision was how long it would be left it. I did not want it to be permanent. That's something that obviously no parent wants. He's got a hole in his throat. Who wants that? And going home with the vent and everything it's scary. The downside to that would be scary other than that there's not really much of a downside." (C7, trach, 1-2 weeks after placement)<br><br>"Just that he wouldn't be able, he wouldn't be able to be a normal little boy and do normal little boy things with a trach…not be able to like run and play." (C15, definitive no trach, 2-3 months since discussion)<br><br>"When we were making the decision, it was the airway emergency, how would I deal with that? Can I keep her safe at home? And that was my—what weighed on me the most." (C11, trach, < 1 week after placement)<br><br>"That's the only thing that I'm scared of having to deal with it [trach] by myself..." (C17, trach, 2-3 weeks after placement)

Supplementary Materials

	"No swimming, no water activities, since we are a beach family... She will still go to the beach. I'm not going to take that away from her either. But just saying she couldn't swim. Not being able to talk right away. I know kids can talk over the trach once they get a little older, and they can…have all that air come up but really not being able to hear her was my downfall…" (C6, trach, 2-3 weeks after placement)<br><br>"Having a nurse, eight hours, sixteen hours a day in my house. I was afraid to…I didn't even set up his crib until they told me he was coming home…I didn't even—before that, a month before that I didn't even know where we were setting up the crib." (C15, definitive no trach, 2-3 months since discussion)<br><br>"We are very secluded so the overall, long-term care for her, distance-wise is definitely going to be a financial strain for us." (C8, trach, 1-2 weeks after placement)<br><br>"A resentment in my kids, my oldest said well you're gonna come home but you're not really gonna be home…when I woke up this morning I thought of how do we go on vacation, like how do we just do a weekend at a hotel like does a nurse come with us? Like how? …my understanding is you need to have eyes on him 24/7…me coming to grips with my ideal with what [patient] childhood will be like… my other kids all got dropped off in nursery during church and built friendships so now I can't just drop him off in the nursery for an hour and a half unless somebody knows how to deal with a trach… like how does he build friendships?" (C13, trach, < 1 week after placement)<br><br>"I would say a downside for some people would be like when people just look at you. It'll be the shame…cause some people are not that strong, so they don't think about what other people think you. But for me maybe in the past but for now it's like who cares." (C10, trach, < 1 week after placement)

Supplementary Materials

Table S3. Illustrative Quotes of Parents' Values Driving the Decision (Theme 3)	
Best interest of child (proof of need and tolerance of tracheostomy)	"She [a relative who knows something about trach] just couldn't wait to talk to me about it [trach] and I was like no I'm not ready yet because it's not there, we're not there yet it hasn't happened and in fact it was postponed from Monday to yesterday so I, until it actually happened I was still resisting it and open to what was going to happen like was he gonna was something cause like things change all the time." (C13, trach, < 1 week after placement)<br><br>"I still have high faith in her even though she has that she's not going to have it forever. It's just not the plan for her right now. The plan was for her to get a trach." (C6, trach, 2-3 weeks after placement)<br><br>"And I'm like wow I have to make this decision and I had to put away my fear and think about the comfort…and the best for my son…so I'm like it's something that I didn't want, but it's something that he needed because he can't go around with a tube down his throat." (C10, trach, < 1 week after placement)<br><br>"A more normal life, and less scary life, especially before this was we started this process, was the tradeoff for her being able to breathe and live." (C11, trach, < 1 week after placement)<br><br>"If I'm gonna have a medically fragile child, and I'm going vow to give them the best life and I'm gonna do within reason everything to give them that. So comfort care was never really an option for me." (C9, trach, < 1 week before placement)
Benefits of trach outweigh its downsides	"So I had to give up my ideal of what I felt like [patient]'s life should look like. I had to give up my idea of what our family will look like… I gained an end date of time of like you can go home now so the time span in the hospital shortened by saying yes to the trach so I gained that pretty much assurance that were not gonna be living here next year." (C13, trach, < 1 week after placement)<br><br>"Being a mom, you don't want to see your child suffering. And getting her tracheostomy, the pros to it outweighed the cons medically." (C8, trach, 1-2 weeks after placement)<br><br>"There's a lot more superficial downsides than there are pros, but it's more so just society I guess. There's infection with the trach there is a slightly higher chance you can get an infection from germs and colds. My biggest con is that it's just me, so then it's you have to have a second person to be trained, so me personally I had to ask my mom, but you always have to have somebody else…" (C9, trach, < 1 week before placement)<br><br>"I felt like that this would help him in the long run. Like this may be like a bump in the road, but it's a possibility he won't need it for the rest of his life. And that he will grow up and be a normal kid. So, I feel like he will live a long life and this was best for him at the end." (C16, trach, 3 months after placement)<br><br>"It would be different if he was in pain or suffering or something like that. Or…if he was gonna be a vegetable. Other than that, he could have a pretty normal life

	with a trach in… I want him to have a long happy life, even if he has to have a trach or even if he's paralyzed or anything like that. Of course, him being able to grow up, that's all I really want for him." (C7, trach, 1-2 weeks after placement)<br><br>"I would never want [patient] like if it came down to [patient] and she's intubated for weeks at a time and things are failing, that's not something that I would ever put her through. If I don't truly think with all the research I've done, second opinions from other doctors, if I don't truly think that she's going to make an improvement or make an improvement that is back to her way of normal life, then we wouldn't have gone through with trach. I don't do anything unless, I'm all for quality over quantity." (C9, trach, < 1 week before placement)
Benefits of trach outweigh other options	"There was a lot that just being intubated was definitely not worth it. Having a trach, he's able to eat and to drink he's able to talk he's able to do physical therapy, get his arms and his legs working. [With] intubation, he can't do none of that." (C7, trach, 1-2 weeks after placement)<br><br>"[Jaw distraction] didn't seem like as good of an option and it's just going and there was a chance she would still need a trach anyway. So it just didn't make sense to do that to her right now." (C11, trach, < 1 week after placement)<br><br>"Upside of just the trach, probably less pain from all of the other surgeries, less complications. I mean there's all sorts of things that could arise from multiple surgeries." (C12, trach in OR, 1-2 weeks after placement)<br><br>"After seeing her last time extubated, in her room and watching that play out and how she reacted when trying to breathe on her own. It was her lungs are great. It's just her airways. Her stomach was just trying to breathe, but it wasn't she's having a hard time and that was it for, I wasn't gonna put her through that again. 3rd time, that's three 3 times." (C6, trach, 2-3 weeks after placement)<br><br>"We personally do not believe in terminating life once it's here." (C8, trach, 1-2 weeks after placement)<br><br>"Just comfort and peace with him in any way we can get it…mainly just try to avoid painful situations or try to whatever, will make him not so miserable in life cause he's already had so much." (C14, pending/no trach yet, ongoing discussion)
Better quantity and quality of life	"My outlook is her quality of life. Definitely, she has shown, she wants to live. She has thrive to—as sick as she is to overcome every negative that the nurses or the doctors have said, well, we don't see her doing this and she has proved them wrong every single time." (C8, trach, 1-2 weeks after placement)<br><br>"I don't see how I can separate those two [quality of life, quantity of life]. I just don't know. They're both equally important and in order to have a quality or a quantity you have to have the quality." (C12, trach in OR, 1-2 weeks after placement)

Supplementary Materials

	"I think quality and quantity went, they were kind of together because if we didn't do something, she...she just, I don't know that she would have survived very long because it was pretty significant." (C11, trach, < 1 week after placement)<br><br>"I think both ways [quality of life, quantity of life] it played a role in thinking of him with a stable airway and being able to get out of the hospital and then also not wanting it so that he could just what he wouldn't be able to leave…so it's the quality of life of coming home and being the little boy that he was created to be." (C13, trach, < 1 week after placement)<br><br>"Quality of life is what's most important. I'd rather him have one day of an awesome life than twenty years of laying in a hospital. So it was difficult because that's what they had said with the trach is—he'd be able to go home and be able to, you know, eat, drink all the stuff he hadn't done." (C15, definitive no trach, 2-3 months since discussion)
